# Patterns of changes in serum lipid profiles in prediabetic subjects: results from a 16-year prospective cohort study among first-degree relatives of type 2 diabetic patients

**DOI:** 10.1186/s12944-020-01371-y

**Published:** 2020-08-23

**Authors:** Shahla Safari, Masoud Amini, Ashraf Aminorroaya, Awat Feizi

**Affiliations:** 1 Isfahan Endocrine and Metabolism Research Center, Isfahan University of Medical Sciences, Isfahan, Iran; 2 Department of Biostatistics and Epidemiology, School of Health, Isfahan University of Medical Sciences, Isfahan, Iran

**Keywords:** Type 2 diabetes, Prediabetes, Triglycerides, Cholesterol, Cholesterol HDL, Cholesterol LDL, Latent Markov model

## Abstract

**Background:**

Lipid abnormality pervasively is associated with the risk of type 2 diabetes mellitus. To the best of our knowledge, there is no study that has examined the longitudinal changes in a wide range of serum lipid profiles in prediabetic subjects in association with the risk of developing type 2 diabetes mellitus in the future. This study aimed to identify the patterns of changes in lipid profiles over time in prediabetic patients and to classify these subjects in order to highlight which patients are at high risk for future diabetes.

**Methods:**

This prospective 16-year (2003–2019) cohort study was conducted among 1228 prediabetic subjects. The study subjects were followed, and the changes in their lipid profiles, including triglycerides, cholesterol, high-density lipoprotein cholesterol, and low-density lipoprotein cholesterol, were evaluated. The latent Markov model was used for data analysis.

**Results:**

The mean (standard deviation) age of subjects was 44.0 (6.8) years, and 73.6% of them were female. The latent Markov model identified two latent states of subjects in terms of changes in lipid profiles: a low tendency to progress diabetes / high tendency to progress diabetes (74, 26%). The latent Markov model showed that the transition probability from a “low tendency to progress diabetic” state to a “high tendency to progress diabetic” state was lower than the transition probability from “high tendency to progress diabetic” state to “low tendency to progress diabetic” state.

**Conclusions:**

The present study showed that more than half of the first-degree relatives of T2DM had approximately normal lipid profiles and that these patients are more inclined to transition from a higher- to a lower-tendency diabetic state. These findings confirm the value of regular screening of first-degree relatives of T2DM. Moreover, preventive intervention strategies are recommended to reduce their risk of developing T2DM.

## Introduction

Type 2 diabetes mellitus (T2DM) is a common chronic disease with major morbidities and high mortality rate [1]. The World Health Organization (WHO) estimated that the number of diabetic people in the world to reach 522 million by 2030, of whom 439 million will have T2DM [2, 3]. The prevalence of diabetes among Iranians was 7.9% in 2010, but the distribution of this prevalence in Iran is also diverged widely between 1.3 and 14.5% in various provinces [4]. Prediabetes (PD) is the precursor stage of diabetes mellitus, in which the subject’s plasma glucose is higher than normal but lower than diabetes mellitus thresholds [5]. In recent years, PD prevalence has increased, especially in developing countries, where PD prevalence is higher than that of T2DM [6]. It is estimated that 5–10% of subjects with PD will develop T2DM [7].

The numerous comorbidities, including obesity and lipid abnormality, are associated with the risk of developing diabetes [8]. Previous evidence suggest that lipid abnormalities are common in people with T2DM and PD [9, 10]. For instance, a meta-analytic review demonstrated that lipid profile disorders are significantly associated with T2DM [11]. A community-based cross-sectional survey showed a strong association between serum lipid profiles with T2DM and PD [12]. Prediabetic individuals often exhibit an atherogenic pattern of risk factors that include high levels of total cholesterol (CHOL), low-density lipoprotein cholesterol (LDL), and triglycerides (TG) and low levels of high-density lipoprotein cholesterol (HDL). Lipid abnormalities in diabetic patients are typically characterized by high CHOL, high TG, low HDL, and high LDL levels [13]. Although the association between lipid abnormality and T2DM has been investigated in various populations, few studies have been conducted to evaluate this association in prediabetic patients as a high-risk population.

Due to the increased risk of diabetes progression over the 5–10 years following the onset of the PD stage [14–16], it is important to establish appropriate prevention strategies in PD. One’s lipid profile is not necessarily stable, especially in prediabetics; accordingly, it is necessary to apply an appropriate analytical technique that can provide a comprehensive evaluation of subjects based on changes in their lipid profiles over time. Therefore, in the current study, an advanced statistical method (i.e., the latent Markov model (LMM)) was used for addressing the above points. The previous studies have described the association between lipid profiles with diabetes, without exploring the patterns of changes in lipid profile over time. LMM, a latent state-switching approach, offer a straightforward approach to classify subjects (latent state) according to the patterns of changes in lipid profile over time. The application of this method results in the identification of subjects within each latent state who are highly similar to each other and uniquely different from those in other states. The LMM estimates the probability to move between the states or to remain in the same state. Subjects were assigned to the latent states which they had the highest probability for membership. This study aimed to identify the pattern of changes in lipid profile over time in prediabetic patients and classify these subjects in order to highlight the high risk people for future diabetes risk.

## Materials and methods

### Study design and participants

The current study was conducted under the framework of the Isfahan Diabetes Prevention Study (IDPS), which was initiated in 2003 among 3483 first-degree relatives (FDRs) from a consecutive sample of patients with T2DM. The IDPS is an ongoing longitudinal study carried out in a cohort of the FDRs of patients with T2DM in Isfahan, which is the largest city in central Iran, to assess the various potential risk factors for diabetes in subjects with a family history of T2DM. The sample of FDR was recruited between 2003 and 2018 and followed up on until 2019. Recruitment methods and examination procedures have been described elsewhere [17]. Prediabetes was defined as: IFG (FPG: 5.6–6.9 mmol/l), and 2-h plasma glucose < 7.8 mmol/l, or IGT (FPG < 7.0 mmol/l, but with 2-h plasma glucose concentration of ≥7.8 mmol/l and < 11.1 mmol/l).

Subjects with T2DM and normal conditions were excluded at the baseline. They completed laboratory tests, including a standard 75 g 2-h oral glucose tolerance test (OGTT), and a questionnaire that asked them about their health status and various potential risk factors of diabetes. Of the 3483 subjects who participated at the baseline, 1228 had been diagnosed with PD. The data from 1228 prediabetics, all of whom had at least two visits during the follow-up period, were used. Data included the baseline measurement, the last measurement, and the mean of the measurements, which was recorded among baseline and last, as the second of measurement.

Full family history was obtained, and physical examinations were performed on all subjects. The standardized blood pressure was measured; also, various potential related risk factors were recorded for prediabetes, diabetes, and high blood pressure. The subjects received follow-up tests according to the Standard of Medical Care in Diabetes [18] to update information regarding lifestyle, anthropometric, and demographic characteristics, as well as to collect data on the diagnosis of prehypertension, hypertension, prediabetes, and diabetes, in every year. Written informed consent was obtained from all subjects in IDPS. The current secondary study has been approved by Bioethics Committee of Isfahan University of Medical Sciences (IR.MUI.MED.REC.1398.532).

### Laboratory parameters

Biochemical tests including lipid profile, fasting plasma glucose (FPG) and OGTT were carried out for all subjects. To determine lipid profile and FPG, a blood sample was drawn from all subjects after 10–12 h of overnight fasting. Postprandial plasma glucose was measured using venous blood sample at 30, 60, and 120 min after oral glucose administration. Plasma glucose and lipid profile concentrations were determined using enzymatic colorimetric method (ParsAzmoon, Tehran, Iran) adapted Selectra-2 auto-analyzer (Vital Scientific, Spankeren, Netherlands). Serum concentration of LDL was calculated by Friedwald equation in subjects with serum TG levels < 400 mg/dL [19]. Serum concentration of HDL, CHOL and TG were measured using standardized procedures [19].

Definitions and diagnostic criteria were based on the American Diabetes Association (ADA) guidelines. Symptomatic subjects with FPG ≥11.1 mmol/L were considered diabetic. If FPG was ≥7 and < 11.1 mmol/L, a second FPG was measured on another day. If the second FPG was also ≥7 mmol/L, subjects were classified as diabetic. FPG ≥7 mmol/L or 2-h PG ≥11.1 mmol/L also defined diabetes mellitus. Impaired glucose tolerance (IGT) was interpreted as 7.8 ≤ 2hpost glucose load (75 g glucose) ≤11.0 mmol/L [5]. Impaired fasting glucose (IFG) was interpreted as 5.6 ≤ FPG ≤ 6.9 mmol/L [5]. In addition, all subjects developing IFG and IGT were pooled in a unique “impaired glucose metabolism” (IGM) group in the analyses.

In the analyses, the following categories were considered as abnormal: TG level of more than 150 mg/dL; LDL level of more than 100 mg/dL and CHOL level more than 200 mg/dL; in both men and women [20], HDL level of less than 40 mg/dL in men; less than 50 mg/dL in women [20]. HDL level > 60 mg/dL, optimal condition, considered protective against heart disease [20].

### Other variables

The subjects completed a demographic questionnaire including age and gender. Physical activity was recorded in an International Physical Activity Questionnaire) IPAQ) [21]. Anthropometric and clinical measurements, including body mass index (BMI) (by dividing weight [kg] to the square of height [m^2^]), FPG and lipid profile include TG, CHOL, HDL and LDL was recorded. The process of administering and collecting the questionnaires was conducted at the Isfahan Endocrine and Metabolism Research Center, Isfahan University of Medical Sciences.

### Statistical analysis

Continuous and categorical basic characteristics of study subjects were presented as mean (standard deviation (SD)) and frequency (percentage), and compared between study groups using analysis of variance (ANOVA) or independent samples T-Test and Chi-square tests, respectively.

To analysis the patterns of changes in serum lipid profile over time in prediabetic patients, LMM was applied. LMM is a hybrid model from the latent class model (LC), and Markov (MC) chain model. LMM may be seen as an extension of the LC in which subjects are allowed to move between the latent classes during the period of observation. These models assume the existence of a latent process which affects the distribution of the response variable. The main assumption behind this approach is that the response variables are conditionally independent given this latent process, which follows a Markov chain with a finite number of states. LMM estimate patient’s condition using latent variables, adjusting for the measurement error contained in the diagnostic tests studied (lipid profiles).

Three measures of lipid indices obtained from subjects including first measure at baseline, mean values during follow up period and last measure have been used. LMM identified number of latent states in studied subjects based on patterns of changes in study lipid measures, also provided the probability moving between various states. The process of LMM fitting was as follows: the following LMM were estimated, 2-State 1-Class, 2-State 2-Class, 2-State 3-Class, 3-State 1-Class, 3-State 2-Class and 3-State 3-Class sequentially, based on lipid profile; TG, CHOL, HDL and LDL responses in prediabetic patients and the model with 2-State 1-Class was selected based on goodness of fit criteria and more interpretability.

The balance between fit and parsimony (number of parameters) of different models was estimated using Akaike Information Criterion (AIC) and the Bayesian Information Criterion (BIC). The optimum number of states was determined through comparing the AIC, BIC, classification error and entropy indices across models. Lower AIC, BIC and classification error and higher entropy, indicate the better model fitting and the better states separation [22, 23]. Two latent states were extracted from TG, CHOL, LDL and HDL in order to evaluate their association with progress diabetes over time, and were labelled as “State1” and “State2”.

After selecting the number of proper latent states, the LMM without/with covariates including age, smoking, gender, physical activity, BMI, and FPG were fitted and the patterns of changes in serum lipid profile over time in prediabetic patients was evaluated. The fitted model was adopted separately in gender subgroups. The interpretation of extracted latent states was done based the mean values of lipid profiles calculated by LMM.

LMM also allows estimating the longitudinal change in the metabolic condition of prediabetic subjects. The LMM estimates the initial and transition probabilities according to the mean values of TG, CHOL, LDL and HDL. The initial probabilities define as the probability of current state that is the one needed to predict the future. The transition probability is defined as, probability to move of subjects between different latent states. The subjects in the PD state can remain and/or move to other latent states. The proposed model was fitted using the LMest package developed within the R free statistical Software (version 3.6.3) [23].

## Results

General characteristics of subjects at baseline are presented in Table 1. Of 1228 study subjects with mean (SD) 44.0 (6.8) years, 73.6% were females. Basic characteristics; smoking (*P* < 0.001), BMI (*P* < 0.001), FPG (*P* < 0.001), Physical activity (*P* < 0.05), TG (*P* < 0.05), and HDL (*P* < 0.001) were statistically significantly different between male and female (Table 1).

**Table 1:** Basic demographic and clinical characteristics of prediabetic subjects at the baseline

Variables	Male(*n* = 324)	Female(*n* = 904)	Total(*n* = 1228)	*P Value*
Age (years)	44.1 ± 7.0	43.9 ± 6.8	44.0 ± 6.8	0.577
Smoking	34(10.5)	9(1)	43(3.5)	< 0.001*
BMI (kg/m^2^)	28.21 ± 3.10	29.54 ± 4.30	29.20 ± 4.07	< 0.001
FPG (mmol/l)	106.20 ± 9.25	103.72 ± 10.16	104.37 ± 9.99	< 0.001
Physical activity (min/week)	36.52 ± 67.94	48.52 ± 79.92	45.35 ± 77.10	0.016
SBP (mmHg)	11.73 ± 1.13	11.61 ± 1.41	11.65 ± 1.34	0.29
DBP (mmHg)	7.79 ± 0.92	7.62 ± 0.92	7.66 ± 0.92	0.35
TG (mg/dl)	197.96 ± 142.44	158.98 ± 84.38	169.84 ± 105.17	0.002
CHOL (mg/dl)	198.95 ± 43.93	197.96 ± 142.44	200.02 ± 40.54	0.706
HDL (mg/dl)	40.13 ± 10.98	47.00 ± 11.80	45.08 ± 11.97	< 0.001
LDL (mg/dl)	121.23 ± 38.45	122.34 ± 33.72	122.03 ± 35.06	0 .376

Values are mean ± SD for continuous and frequency (percentage) for categorical variables. BMI (Body mass index), FPG (Fasting blood glucose in the baseline), SBP (systolic blood pressure), DBP (diastolic blood pressure), CHOL) total cholesterol), LDL (low-density lipoprotein cholesterol), TG) triglycerides), HDL (high-density lipoprotein cholesterol), T- Test, Chi-Square test *, *P Value* < 0.05 is considered as significant

General characteristics of subjects at the end of follow up are presented in Table 2. Over the 16-year follow-up, 339(27.6%) became diabetic, 204(16.6%) was normal, 403 (32.8%) remained PD (IFG and IGT) and the data about final status of 282 (23%) of subjects was not available. The mean age (*P* < 0.05) and FPG (*P* < 0.001) was significantly different between study groups, also a significant gender distribution was observed (*P* < 0.05).

**Table 2:** Basic demographic and clinical characteristics of different categories of subjects at the end of follow-up

Variables	Pre diabetic					*P Value*
	IFG (*n* = 303)	IGT (*n* = 100)	IGM (*n* = 403)	NGT(*n* = 204)	DM (*n* = 339)	
Age (years)	43.8 ± 7.1	42.1 ± 5.2	43.4 ± 6.7	42.9 ± 6.3	44.5 ± 6.8	0.021*
(Male/Female)(%)	(7.0/ 25.1)	(2.4/ 8.1)	(9.4/ 33.2)	(7.2/ 14.4)	(9.5/ 26.3)	0.011**
BMI (kg/m^2^)	29.13 ± 3.74	28.96 ± 3.64	29.08 ± 3.71	28.84 ± 4.26	29.60 ± 4.35	0.150*
FPG (mmol/l)	105.18 ± 8.56	97.92 ± 9.67	103.37 ± 9.37	101.12 ± 8.63	106.20 ± 11.16	< 0.001*
Physical activity (min/week)	49.16 ± 81.29	63.36 ± 98.79	52.68 ± 86.05	46.02 ± 79.08	54.08 ± 72.11	0.498*

Values are mean ± SD for continuous and frequency (percentage) for categorical variables. IFG (impaired fasting glucose), IGT (impaired glucose tolerance), IGM (impaired glucose metabolism, including subjects with IGT and/or IFG), NGT (normal glucose tolerance), DM (diabetes group), BMI (Body mass index), FPG (Fasting blood glucose in the baseline), ANOVA test *, Chi-Squared test**, *P Value* < 0.05 is considered as significant

A series of LMM was estimated to determine the number of latent states (2-3State 1-3Class). All models’ fit criteria as well as interpretability of extracted states strongly suggested a LMM with two latent states based on the patterns of changes in lipid profile. According to fit criteria, 2-State 1-Class model selected, with lower AIC, BIC, parameter number and classification error and as well as higher entropy (Table 3).

**Table 3:** Model fitting criteria for prediabetic subjects by latent Markov analysis

Fitted models	Log Likelihood	BIC	AIC	Number of parameter^a^	Classification. Error	Entropy R-squared
2-State 1-Class	−54,789.4762	109,714.1020	109,616.9523	19	0.0920	0.6052
2-State 2-Class	−54,782.2414	109,728.0850	109,610.4828	23	0.0944	0.5924
2-State 3-Class	−54,782.1874	109,756.4296	109,618.3748	27	0.0944	0.5925
3-State 1-Class	−53,737.2923	107,702.2051	107,538.5846	32	0.1421	0.6681
3-State 2-Class	−53,722.8693	107,737.3773	107,527.7385	41	0.1412	0.6695
3-State 3-Class	−53,717.2883	107,790.2337	107,534.5766	50	0.1427	0.6656

^a^Parameters are those unknown quantities that are estimated during the fitting of LMM

Table 4 presents the results of fitting the LMM regarding the identified latent states of subjects based on the means of lipid profiles of the total sample, as well as the male and female samples. For total, males, and females samples, two latent states were identified. The interpretation of states is based on the mean of the lipid profiles. The state1 consists of subjects who had relatively few problems in their lipid profile levels; that is, the subjects in this state had relatively low lipid profile values. This state reflects a low tendency to progress diabetes in the future and consists of 74, 74, and 69% of the samples. The subjects in this state had normal TG and CHOL levels. TG levels in males were borderlines, and LDL levels in all groups were borderlines. The state2 consists of subjects who had relatively many problems in their lipid profile levels; that is, the subjects in this state had relatively high lipid profile values. This state indicates a high tendency for diabetes to progress in the future and consists of 26, 26, and 31% of the samples. The subjects in this state had abnormal levels of TG, CHOL, and LDL. HDL levels were normal in males and overall, and they were borderlines for females. Similar findings were observed when LMM was fitted separately in male and female genders.

**Table 4:** The identified latent states of prediabetic subjects based on lipid profile resulted from latent Markov analysis

Group	Lipid profile	Levels of diabetes tendency		Levels of diabetes tendency	
		(without covariates)		(with covariates)	
		Low (State1)	High (State2)	Low (State1)	High (State2)
Total(n=1228)	State size	0.7430	0.2570	0.7345	0.2655
	TG	142.9682	265.1101	142.5925	262.1813
	CHOL	192.1266	227.3209	191.6593	227.4827
	HDL	44.9375	44.7703	44.9247	44.8123
	LDL	117.2795	133.8731	116.8747	134.5226
Males(n=324)	State size	0.7355	0.2645	0.7404	0.2596
	TG	159.8346	307.1037	160.0979	296.2107
	CHOL	191.4662	216.0068	191.2984	216.4241
	HDL	39.7675	41.1365	39.7413	41.2079
	LDL	117.7630	121.8403	117.5796	122.4382
Females(n=904)	State size	0.6916	0.3084	0.6833	0.3167
	TG	133.7960	240.4687	133.5538	238.1966
	CHOL	190.4004	229.3418	189.8000	229.6404
	HDL	46.3990	47.1298	46.3120	47.3042
	LDL	116.1769	136.4491	115.6906	137.0170

The covariates include: age, smoking, gender, BMI, FPG and Physical activity

Table 5 presents the initial and transition probabilities observed during the study from one particular state to other states. Based on the estimates, at the beginning of the period of investigation, in all groups (i.e., total, males, and females), more than half (initial probabilities were 0.73, 0.74, and 0.68) of subjects were in the latent state (i.e., state1). The probability of being in state1 (with or without covariates) is higher than that of being in state2. According to these results, a subject in state1 or in state2 will likely remain at the same condition (without/with covariates), with a probability ranging from 0.78 to 0.97. In all groups, the probability of transitioning from state1 to the state2 is lower than the probability of transitioning from state2 to state1.

**Table 5:** Initial and transition’s probabilities of prediabetic subjects resulted from latent Markov analysis

Group	Status	Levels of diabetes tendency		Levels of diabetes tendency	
		(without covariates)		(with covariates)	
		Low (State1)	High (State2)	Low (State1)	High (State2)
Total (n=1228)	State0	0.73	0.27	0.73	0.27
	State1	0.94	0.06	0.94	0.06
	State2	0.22	0.78	0.23	0.77
Males(n=324)	State0	0.74	0.26	0.74	0.26
	State1	0.96	0.04	0.96	0.03
	State2	0.10	0.90	0.11	0.89
Females(n=904)	State0	0.68	0.32	0.68	0.32
	State1	0.92	0.08	0.91	0.08
	State2	0.22	0.78	0.23	0.77

The state0 is initial state. Probabilities represent the probability of transition from a particular state to other states from row to column

## Discussion

In this prospective longitudinal study, 1228 prediabetic patients were followed from 2003 to 2019, and changes in their serum lipid profiles were evaluated over time using the LMM. Two latent states were identified based on the patterns of changes in lipid profiles’ mean values and the states characterized by levels of the tendency for diabetes to progress (low/ high), with prevalence rates of 74 and 26%, respectively.

The lipid profile mean of subjects assigned to the “high tendency to progress diabetes” state was higher than that of subjects assigned to the “low tendency to progress diabetes” state. The probability of transitioning from the low- to the high-tendency state was lower than the probability of transitioning probability from the high- to the low-tendency state.

No previous study has classified prediabetic patients into homogeneous states based on lipid profile means over time using LMM. However, there are many related studies that have examined the general population, as well as some specific populations, by applying simple statistical methods [8, 9, 24, 25].

Previous studies have focused on investigating the association of each lipid profile (i.e., TG, CHOL, HDL, and LDL) with the risk of developing diabetes in the future or concurrently, separately. For instance, in Framingham Heart’s [26] study, T2DM subjects were compared with those without T2DM; they had higher plasma TG levels and lower HDL levels. In a case-control study, Vineetha et al. documented statistically significantly higher TG values for and lower HDL values in subjects with PD [27].

It is believed that lipid profile abnormality is a strong risk factor for T2DM among prediabetic patients [16, 28]. In the present study, the subjects with a high tendency to progress their diabetes state had lipid profile abnormalities. The mean of lipid profile abnormality was associated with “high/low tendency to progress diabetes” states. This finding is in line with the results of previous studies which have emphasized the association of lipid profile disorders with the risk of diabetes [10, 11, 29, 30].

Bhowmik et al. obtained similar results as the current study in terms of the levels of dyslipidemia. Their results showed a strong association between serum lipid profile and T2DM and PD. In addition, high levels of TG in combination with low levels of HDL showed the highest association with T2DM and PD. High CHOL, high TG, and low HDL levels were more prominent among subjects with T2DM and PD [12].

In the present study, in an irregular pattern, low HDL levels were not associated with increased T2DM. In line with the present study, Hasse et al. reported that genetically reduced HDL was not associated with increased T2DM, suggesting that the corresponding observational association is due to confounding and/or reverse causation [8]. In contrast, in the Hawaii-Los Angeles-Hiroshima study, Hirano found that HDL is a predictor of T2DM for Japanese-American and native Japanese people independently of age and gender [31]. In a population-based longitudinal survey conducted on a cohort of high-risk individuals in Iran, Janghorbani et al. showed that low HDL levels are a weak predictor of T2DM independently of age and gender [32].

Although numerous research works have examined the risk factors of diabetes, most research has ignored the complexity of diabetes and the reversibility of diabetic states. In the current study, the probability with a low tendency to progress to diabetic state and to remain in the same condition, in the long run, is greater in comparison to the ones with a high tendency to progress to diabetes. Further, the probability of transitioning from low tendency to a high tendency was lower than the probability of transitioning in the opposite direction.

### Study strengths and limitations

A major strength of the present study is its application of LMM for classifying subjects according to patterns of changes in lipid profiles over time instead of considering them as a single index. Other strengths of this study are its population (which consisted of a large cohort of prediabetic patients), the length of the follow-up period of these subjects (16 years), and adjustments for some potential confounders in the analyses. The current findings were drawn from a study population of prediabetic patients; therefore, the results may not be applicable to all populations. In this study, some potentially useful information, such as heart disease and some lifestyle variables were not included.

## Conclusions

The present study showed that more than half of PD involves normal CHOL and TG levels and borderline LDL and HDL levels (state1). Also, these patients are more likely to transition from a higher to a lower tendency to enter a diabetic state. These findings confirm the value of regular screenings of FDR with T2DM. Preventive intervention strategies are recommended to reduce patients’ chances of developing T2DM.

## Data Availability

The data that support the findings of this study are available from the corresponding author upon reasonable request.
